# Kératoses séborrhéiques géantes de siège inhabituel

**DOI:** 10.11604/pamj.2017.28.72.13766

**Published:** 2017-09-25

**Authors:** Ilhame Naciri, Nadia Ismaili

**Affiliations:** 1 Service de Dermatologie et Vénérologie, Centre Hospitalier Universitaire IBN Sina, Faculté de Médecine et de Pharmacie, Université Mohammed V, Rabat, Maroc

**Keywords:** Kératose séborrhéique, géante, siège inhabituel

## Abstract

Seborrhoeic keratoses (SK) are benign tumors that mainly occur in the head and in the trunk. Their size generally varies from a few millimeters up to a few centimeters. Giant lesions are very rare but pose a problem in terms both of treatment and transformation. Their occurrence in the genital area is even more rare, posing a problem in differential diagnosis of genital warts. Differentiation is only based on histopathology. We report the case of a 80-year old man, with no particular past medical history, who presented with asymptomatic abdominal and genital lesions evolving over the last twenty years. Clinical examination showed four hyperpigmented, humped, well-defined tumors, with warty surface measuring 2-10 cm along its longer axis (A). Dermoscopic examination showed cerebriform convolutions appearance suggesting seborrhoeic keratoses (B). Histology confirmed the diagnosis, without signs of viral infection or malignant transformation. The patient underwent surgical resection resulting in permanent scar.

## Image en médecine

Les kératoses séborrhéiques (KS) sont des tumeurs bénignes qui siègent électivement au niveau de la tête et du tronc. Leur taille varie généralement de quelques millimètres à quelques centimètres. Les lésions géantes sont très rares, posant un problème de prise en charge et de transformation, et leur emplacement sur la zone génitale est encore plus rare, posant un problème de diagnostic différentiel avec les condylomes et dont la différenciation est faite uniquement sur l'histopathologie. Nous rapportons le cas d’un homme de 80 ans, sans antécédents pathologiques particuliers, qui consultait pour des lésions abdominales et génitales asymptomatiques évoluant depuis vingt ans. L’examen clinique trouvait quatre tumeurs hyperpigmentées en relief, bien limitées, à surface verruqueuse mesurant entre 2 et 10 cm de grand axe (A). L’examen dermoscopique objectivait un aspect de circonvolutions cérébriformes, évoquant des kératoses séborrhéiques (B). L’histologie avait confirmé le diagnostic sans objectiver de signes d’infection virale ni de transformation maligne. Un traitement par exérèse chirurgicale était entrepris au prix d’une cicatrice indélébile.

**Figure 1:**
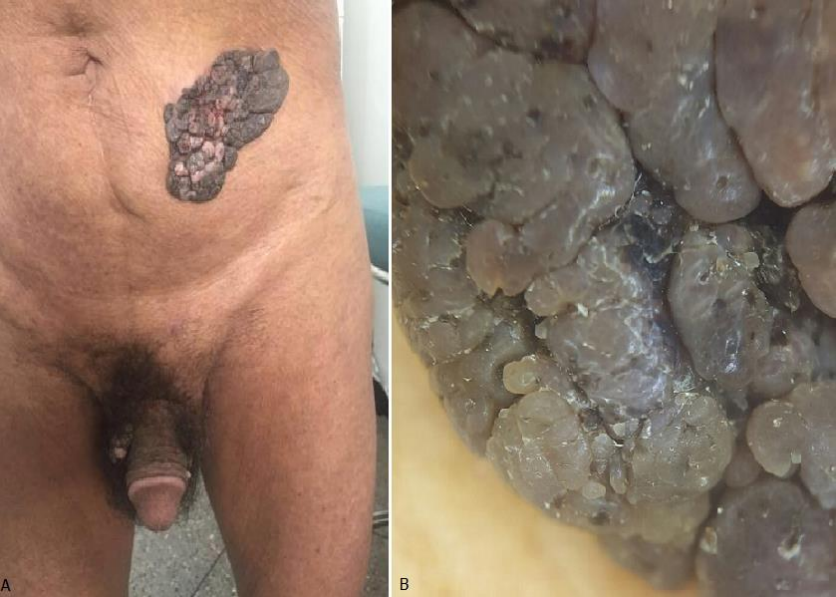
A) lésions hyper pigmentées en relief, siégeaient au niveau génital et abdominal; B) démoscopie, aspect cérébriforme et kystes cornés

